# Effect of lidocaine on kanamycin injection-site pain in patients with multidrug-resistant tuberculosis

**DOI:** 10.5588/ijtld.18.0091

**Published:** 2018-08

**Authors:** R. G. Court, L. Wiesner, M. T. Chirehwa, A. Stewart, N. de Vries, J. Harding, T. Gumbo, H. McIlleron, G. Maartens

**Affiliations:** * Division of Clinical Pharmacology, Department of Medicine, University of Cape Town, Cape Town; † Clinical Research Centre, Faculty of Health Sciences, University of Cape Town, Cape Town; ‡ Brooklyn Chest Hospital, Cape Town; § D P Marais Hospital, Cape Town, South Africa; ¶ Center for Infectious Diseases Research and Experimental Therapeutics, Baylor Research Institute, Baylor University Medical Center, Dallas, Texas, USA

**Keywords:** TB, injectable, adherence, adverse effect

## Abstract

**SETTING:**

Reducing pain from intramuscular injection of kanamycin (KM) could improve the tolerability of multidrug-resistant tuberculosis (MDR-TB) treatment. Lidocaine has been shown to be an effective anaesthetic diluent for some intramuscular injections, but has not been investigated with KM in the treatment of adult patients with MDR-TB.

**OBJECTIVE AND DESIGN:**

We performed a randomised single-blinded crossover study to determine if lidocaine reduces KM injection-site pain. We recruited patients aged ⩾18 years on MDR-TB treatment at two TB hospitals in Cape Town, South Africa. KM pharmacokinetic parameters and a validated numeric pain scale were used at intervals over 10 h following the injection of KM with and without lidocaine on two separate occasions.

**RESULTS:**

Twenty participants completed the study: 11 were males, the median age was 36 years, 11 were HIV-infected, and the median body mass index was 17.5 kg/m^2^. The highest pain scores occurred early, and the median pain score was 0 by 30 min. The use of lidocaine with KM significantly reduced pain at the time of injection and 15 min post-dose. On multiple regression analysis, lidocaine halved pain scores (adjusted OR 0.5, 95%CI 0.3–0.9). The area under the curve at 0–10 h of KM with and without lidocaine was respectively 147.7 and 143.6 μg·h/ml.

**CONCLUSION:**

Lidocaine significantly reduces early injection-site pain and has no effect on KM pharmacokinetics.

## Abstract (FR)

**CONTEXTE:**

Réduire la douleur liée aux injections intramusculaires de kanamycine (KM) pourrait améliorer la tolérance du traitement de la tuberculose multirésistante (TB-MDR). La lidocaine s'est avérée un diluant anesthétique efficace pour certaines injections intramusculaires, mais n'a pas été étudiée pour la KM dans le traitement des patients adultes atteints de TB-MDR.

**OBJECTIF et SCHEMA:**

Nous avons réalisé une étude randomisée croisée en simple aveugle afin de déterminer si la lidocaine réduisait la douleur au site d'injection de la KM. Nous avons recruté des patients âgés de ⩾18 ans sous traitement de TB-MDR dans deux hôpitaux TB du Cap, Afrique du Sud. Les paramètres de pharmacocinétique de la KM et une échelle numérique validée de douleur ont été utilisées à des intervalles réguliers pendant >10 h suivant l'injection de KM avec et sans lidocaine lors de deux occasions distinctes.

**RÉSULTATS:**

Vingt participants ont terminé l'étude : 11 hommes, d'âge médian 36 ans, 11 infectés par le virus de l'immunodéficience humaine et avec un indice de masse corporelle médian de 17,5 kg/m^2^. Les scores de douleur les plus élevés sont survenus tôt et le score de douleur médian a été de 0 à 30 min. L'utilisation de lidocaine avec la KM a significativement réduit la douleur au moment de l'injection et 15 min après. En analyse de régression multiple, la lidocaine a réduit de moitié les scores de douleur (OR ajusté 0,5 ; IC95% 0,3–0,9). L'aire sous la courbe (AUC_0–10_) de la KM avec et sans lidocaine a été de 147,7 et de 143,6 μg·h/ml, respectivement.

**CONCLUSION:**

La lidocaine réduit significativement la douleur précoce au site d'injection et n'a aucun effet sur la pharmacocinétique de la KM.

## Abstract (SP)

**MARCO DE REFERENCIA:**

Disminuir el dolor de la inyección intramuscular de kanamicina (KM) podría mejorar la tolerabilidad del tratamiento de la tuberculosis multirresistente (TB-MDR). Se ha demostrado que la lidocaína es un anestésico eficaz que se puede utilizar como diluyente de algunas inyecciones intramusculares, pero no se ha investigado con la KM en los pacientes adultos tratados por TB-MDR.

**OBJETIVO Y MÉTODO:**

Se llevó a cabo un estudio transversal aleatorizado con anonimato sencillo, a fin de determinar si la lidocaína disminuye el dolor en el punto de inyección de la KM. Se incluyeron en el estudio pacientes de edad de ⩾18 años que recibían tratamiento por TB-MDR en dos hospitales de atención de la TB en la Ciudad del Cabo, Sudáfrica. Se evaluaron los parámetros farmacocinéticos de la KM y se utilizó con intervalos una escala numérica del dolor validada durante 10 h después de la inyección de KM, con y sin lidocaína en dos ocasiones separadas.

**RESULTADOS:**

Veinte participantes completaron el estudio, a saber: 11 hombres con una mediana de edad de 36 años, 11 con infección por el virus de la inmunodeficiencia humana y una mediana del índice de masa corporal de 17,5 kg/m^2^. Las puntuaciones más altas del dolor se observaron temprano y la mediana de la puntuación fue 0 a los 30 min. La adición de lidocaína a la KM redujo de manera significativa el dolor en el momento de la inyección y 15 min después de la dosis. Según el análisis de regresión múltiple, la lidocaína redujo a la mitad las puntuaciones del dolor (OR ajustado 0,5; IC95% 0,3–0,9). El área bajo la curva AUC_0–10_ (de concentración y tiempo) de la KM con y sin lidocaína fue 147,7 y 143,6 μg·h/ml, respectivamente.

**CONCLUSIÓN:**

La lidocaína disminuye de manera significativa el dolor inicial en el punto de inyección y no produce ninguna modificación en la farmacocinética de la KM.

TREATMENT COMPLETION RATES in multi-drug-resistant tuberculosis (MDR-TB) are poor. Only half of patients with MDR-TB are successfully treated.^1^ Treatment default, reported to be as high as 40% in some settings, is a significant contributor to poor treatment outcomes.^2–4^ The drugs used to treat MDR-TB have significant adverse effects, which have been described in one qualitative study to be worse than the disease itself,^5^ and may result in poor treatment adherence or loss to follow-up.^6^

Kanamycin (KM) is a key second-line drug in the intensive phase of treatment for MDR-TB, but has considerable toxicity, including irreversible deafness and renal impairment.^7–9^ The pain associated with the intramuscular administration of KM is also significant and, with repeated dosing, a painful induration may develop at the injection site.^5^Lidocaine is a local anaesthetic which significantly reduces the pain immediately following the intramuscular injection of some drugs,^10^,^11^ but it is currently unknown whether lidocaine has a similar effect in adult patients treated with KM for MDR-TB. Addressing the pain associated with KM administration could enhance the tolerability of MDR-TB treatment regimens and improve long-term outcomes.

## STUDY POPULATION AND METHODS

We performed a randomised single-blinded crossover study to compare injection-site pain from intramuscular KM with and without lidocaine. We recruited patients who were aged ⩾18 years between July 2016 and April 2017 on standard treatment for MDR-TB at Brooklyn Chest Hospital and D P Marais Hospital in Cape Town, South Africa.

During the study period, the treatment regimen for MDR-TB comprised pyrazinamide, moxifloxacin, KM, terizidone, and either ethionamide or isoniazid depending on the results of the line-probe assay for *kat*G and *inh*A Mycobacterium tuberculosis mutations identified in pretreatment sputum cultures, which would indicate high-level resistance to isoniazid or ethionamide, respectively.^12^ Ethambutol (EMB) was added if a patient had no exposure to EMB in the month before treatment initiation and suspicion for EMB resistance was low.^13^ Randomisation was performed manually by an appointed administrator who was not involved in the study design or implementation. Twenty cards, half of which were labelled ‘lidocaine’ and other half labelled ‘no lidocaine’, were placed in separate sealed opaque envelopes before randomisation. Immediately before each participant's first pain assessment, a new envelope was opened revealing whether KM was to be administered alone or mixed with lidocaine. The envelopes were opened sequentially starting with envelope no. 1 for the first participant. As some participants were unable to complete the study and recruitment therefore continued beyond the initial recruitment target, six additional envelopes were prepared in an identical manner. The KM dose was adjusted for creatinine clearance at the discretion of the treating clinician and the same dose was administered on both pain assessment occasions. The Wong-Baker FACES^®^ pain rating scale (Wong-Baker FACES Foundation, Oklahoma City, OK, USA) was used to assess the pain caused by the KM injection on two separate occasions approximately 7–14 days apart.^14^ The Wong-Baker Faces pain rating scale is a validated pain assessment tool designed to assist health care providers measure pain using patient self-assessment. The scale is numbered 0 to 10 and is accompanied by a ‘grimace scale’ of faces to assist patients with the interpretation of pain, whereby the facial grimace increases with higher reported pain scores. We assessed pain at the following time points post-dose: immediately after the injection as well as at 15 min, 30 min, 60 min, 2 h, 6 h and 10 h. Participants were blinded to the addition of lidocaine and to the results of their previous pain assessments to avoid influence from earlier pain scores.

One ml of 2% lidocaine was mixed with a 3 ml ampule of KM to create 4 ml of solution volume. Depending on the prescribed dose of KM, either 2 ml of the solution (500 mg), 3 ml of solution (750 mg) or the entire 4 ml of the solution (1 g) were administered intramuscularly into the superior-lateral quadrant of the buttock using a 22-gauge needle on the opposite side to that used the previous day to prevent the influence of residual pain from the previous dose. If lidocaine was not administered together with KM, participants received either a 3 ml (1 g), 2.25 ml (750 mg) or 1.5 ml (500 mg) KM injection. Blood was drawn on both occasions pre-dose as well as at 2, 4, 6, 8 and 10 h post-dose to assess the effect of lidocaine on KM pharmacokinetics. We used the K10 Anxiety and Depression Scale to screen for psychological distress, which we considered to be a possible influence on participant pain thresholds.^15^ The K10 Anxiety and Depression Scale was specifically designed to identify psychological distress in the previous month using 10 questions. The answers to the questions are scored 1–5, with higher scores recorded with increasing symptom frequency. Patients who score under 20 are likely to be well, with higher scores associated with increasing severity of mental disorders.^16^

Plasma concentrations of KM A were determined using liquid chromatography-tandem mass spectrometry validated according to US Food and Drug Administration and European Medicines Agency guidelines.^17^,^18^ Samples were processed with a solid-phase extraction method using 50 μl of plasma. The extracted sample (5 μl) was injected onto the high-performance liquid chromatography column. Isocratic chromatographic separation was achieved on a Discovery C18 analytical column (5 6 μm, 50 mm 3 4.6 mm) using 4 mM heptafluorobutyric acid in 0.1% formic acid in water/acetonitrile (80:20, *v/v*) at a flow-rate of 500 μl/min. The mobile phase flow was split (1:1) at the source of the mass spectrometer. An AB Sciex API 3000 mass spectrometer (Applied Biosystems, Foster City, CA, USA) was operated at unit resolution in multiple-reaction monitoring mode to monitor transition of the protonated molecular ions at *m/z* 485.2 to the product ions at *m/z* 163.2 for KM A and the protonated molecular ions at *m/z* 494.3 to the product ions at *m/z* 165.3 for the KM-d9 internal standard. Electrospray ionisation was used for ion production. The assay was validated over the concentration range 0.625–40 μg/ml. The combined accuracy (%Nom) and precision (%CV) statistics of the limit of quantification, low, medium and high-quality controls (three validation batches, *n* = 18) were respectively 101.3–107.0%, and 3.0–14.3%.

### Sample size

Using the report by Park et al. describing KM pharmacokinetics,^19^ and assuming that data would be non-parametric, we estimated that a minimum of 16 patients would be required to detect a 20% change in pharmacokinetics between the two groups. Considering the paucity of data on KM pharmacokinetics, we considered a target sample size of 20 participants would be sufficient to allow for any inaccuracy in power estimation.

### Statistics

We used Stata v15.0 (Stata Corp, College Station, TX, USA) to compare pain scores using the Wilcoxon signed-rank test with and without lidocaine at each time point, as well as non-compartmental KM pharmacokinetics with and without lidocaine. We determined the following pharmacokinetic parameters: area under the concentration–time curve at 0–10 h (AUC_0–10_), AUC to infinity (AUC_∞_), half-life, peak concentration (C_max_) and time to C_max_. We used the trapezoidal rule to calculate AUC_0–10_ and extrapolation of the exponential decline to calculate AUC_∞_. In a secondary analysis, we used multilevel ordered logistic regression to identify the factors associated with participant pain scores, including participants who completed the study and those who completed only one pain assessment. We identified the following variables a priori, which we analysed with single regression analyses: the use of lidocaine, time of pain assessment post-dose, human immunodeficiency virus (HIV) status, KM dose in mg/kg, volume of solution injected, sequence of randomisation, body mass index, sex, age and presence of psychological distress, defined as a score of ⩾20 using the K10 screening tool.^16^ We included variables with *P* < 0.2 in the multiple regression analysis.

### Ethics

The study protocol was approved by the University of Cape Town Human Research Ethics Committee, Cape Town (HREC 105/2016). Informed consent was provided by each participant in their language of choice (English, Afrikaans or Xhosa).

## RESULTS

We recruited 29 participants, 20 of whom finished the study, and five completed pain assessments on only one occasion. Characteristics at each dosing occasion of the 20 participants who completed the study are given in Table 1. Twelve participants received 750 mg, five participants received 1000 mg and three participants were dosed with 500 mg of KM. One participant had a reduced creatinine clearance of 30.2 ml/min on the first and 34.3 ml/min on the second dosing occasion.

**Table 1:**
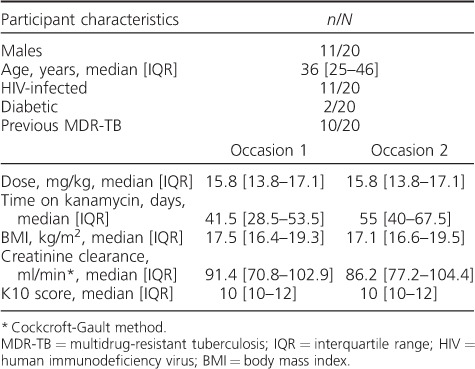
Participant characteristics of 20 patients on treatment for MDR-TB in a single-blinded randomised crossover pharmacokinetic analysis of kanamycin with and without lidocaine

Pharmacokinetic parameters and drug concentrations of KM dosed with and without lidocaine are shown in Table 2; 19 participants were included as the KM concentrations for one participant were not available at the time of data analyses. The Figure shows the pain scores following KM administration with and without lidocaine at each evaluated time point post-dose. Lidocaine dosed with KM reduced the pain scores reported by participants significantly immediately following the injection (*P* = 0.02) and at 15 min after dosing (*P* = 0.02). On multilevel ordered logistic regression analyses (Table 3), two factors were negatively associated with differences in participant pain scores at each dosing occasion: use of lidocaine and time post-dose. HIV infection had a positive association with pain scores. We found symptoms of psychological distress, including depression, in three participants, but there was no association of psychological distress with pain.

**Table 2:**
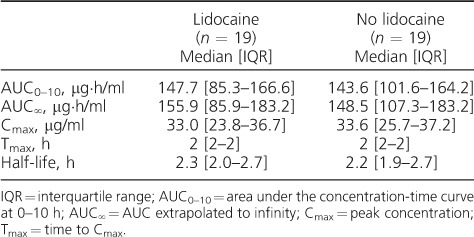
Pharmacokinetic parameters of kanamycin at steady state with and without lidocaine in a crossover study of patients on treatment for multidrug-resistant tuberculosis

**Figure:**
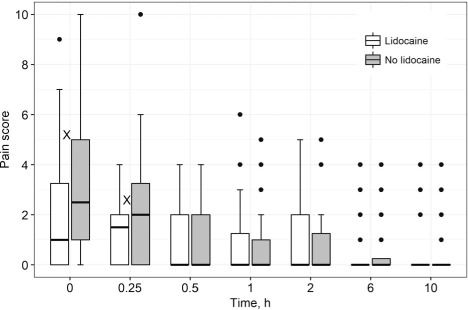
Median pain scores with and without lidocaine at each time point following the intramuscular administration of kanamycin in 20 participants treated for multidrug-resistant tuberculosis. ^X^ P = 0.02. Box midlines = median values; upper and lower bounds of boxes = interquartile range; upper and lower bounds of whiskers = 1.5 × the interquartile range.

**Table 3:**
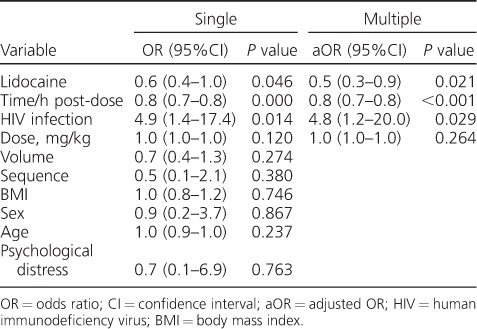
Multilevel ordered logistic regression analysis of the factors associated with injection-site pain from intramuscular administration of kanamycin with and without lidocaine in participants on treatment for multidrug-resistant tuberculosis

## DISCUSSION

Our finding that lidocaine co-administered with KM reduces injection-site pain in patients treated for MDR-TB could potentially improve the tolerability of MDR-TB treatment. KM is currently considered a key component of treatment regimens for MDR-TB by the World Health Organization.^20^ The toxicity and poor tolerability of the drugs used to treat MDR-TB are widely accepted to be significant factors affecting adherence and retention in care, resulting in poor treatment outcomes.^6^ Reducing the pain associated with the intramuscular injection of KM, which is often not included in reports describing adverse effects in patients treated for MDR-TB, is an important step towards improving the tolerability of the intensive phase of MDR-TB treatment.

We found that use of lidocaine significantly lowered pain scores in the first 15 min following KM injection, which is important considering that the highest pain scores were reported by participants immediately post-dose, and that the median time to resolution of pain was 30 min. Lidocaine has a rapid onset of action and a limited toxicity profile unless administered intravenously.^21^,^22^ Furthermore, lidocaine is inexpensive and widely available and therefore appropriate for use in low- and middle-income settings, which have the highest burden of MDR-TB.

Data on KM pharmacokinetics are limited. We found that the C_max_ and AUC of KM were in accordance with the expected range.^19^,^23^ A higher C_max_ was found in a small study of patients treated for MDR-TB in Korea with a higher dose of KM.^24^ Lidocaine had no effect on KM pharmacokinetics; this finding is in line with the findings of others who have assessed the effect of lidocaine on the pharmacokinetics of other drugs when administered intra-muscularly.^10^

In the regression analysis, we found use of lidocaine to be independently associated with pain reduction in the single and multiple variable model. As participants received a higher volume of solution when dosed with lidocaine, we explored the possibility in the regression model that a higher volume of injected solution may increase pain due to a greater stretch on pain receptors, but found this to be non-significant. We found HIV infection to be associated with a higher pain score, although this finding needs to be interpreted with caution as the confidence intervals were wide, likely due to the small sample size. HIV infection has been associated with increased morbidity, including depression, which has been shown in several studies to lower pain thresholds.^25^,^26^ We observed symptoms of psychological distress, including anxiety and depression, in three participants who were HIV-infected, although the presence of these symptoms was not associated with higher pain scores in the regression analysis. However, our study was not powered to assess the influence of mental health or HIV infection on pain scores.

Our study had three main limitations. First, for logistical reasons only the participants were blinded to whether KM was dosed with lidocaine or not. However, single blinding was very unlikely to cause bias as the participants completed the pain scores without input from study staff. Second, pain assessments were done on occasion 1 and occasion 2 after a median of approximately 6 and 8 weeks of treatment, respectively. As repeated administration of KM may result in a painful induration at the injection site, we cannot exclude the possibility that pain scores may have been different if pain assessments had been conducted sooner or later after treatment initiation. Third, study participants were all in-patients with a high prevalence of comorbidities and psychosocial problems. The results may therefore not be generalisable to all MDR-TB patients.

## CONCLUSION

Lidocaine use reduces the pain associated with KM injections. Given the toxicity and poor tolerability of the MDR-TB treatment regimen, reducing the pain caused by the injectable during the intensive phase of treatment could potentially result in improving adherence and, ultimately, treatment completion rates in MDR-TB.
